# Supplementary material for: Risk factors for sacrococcygeal pilonidal sinus: a systematic review and meta-analysis supplemented by genetic causal assessment
Source: Front Surg. 2026 Jan 7;12:1718589. doi: 10.3389/fsurg.2025.1718589 (PMC12819706; doi:10.3389/fsurg.2025.1718589)
Supplement: Supplementary file 2 [file Datasheet2.zip › Supplementary Data 2/MR_pipeline_p5e-8/funnel_plot/ukb-d-1747_6.finngen_R12_L12_PILONIDALCYST.pdf]

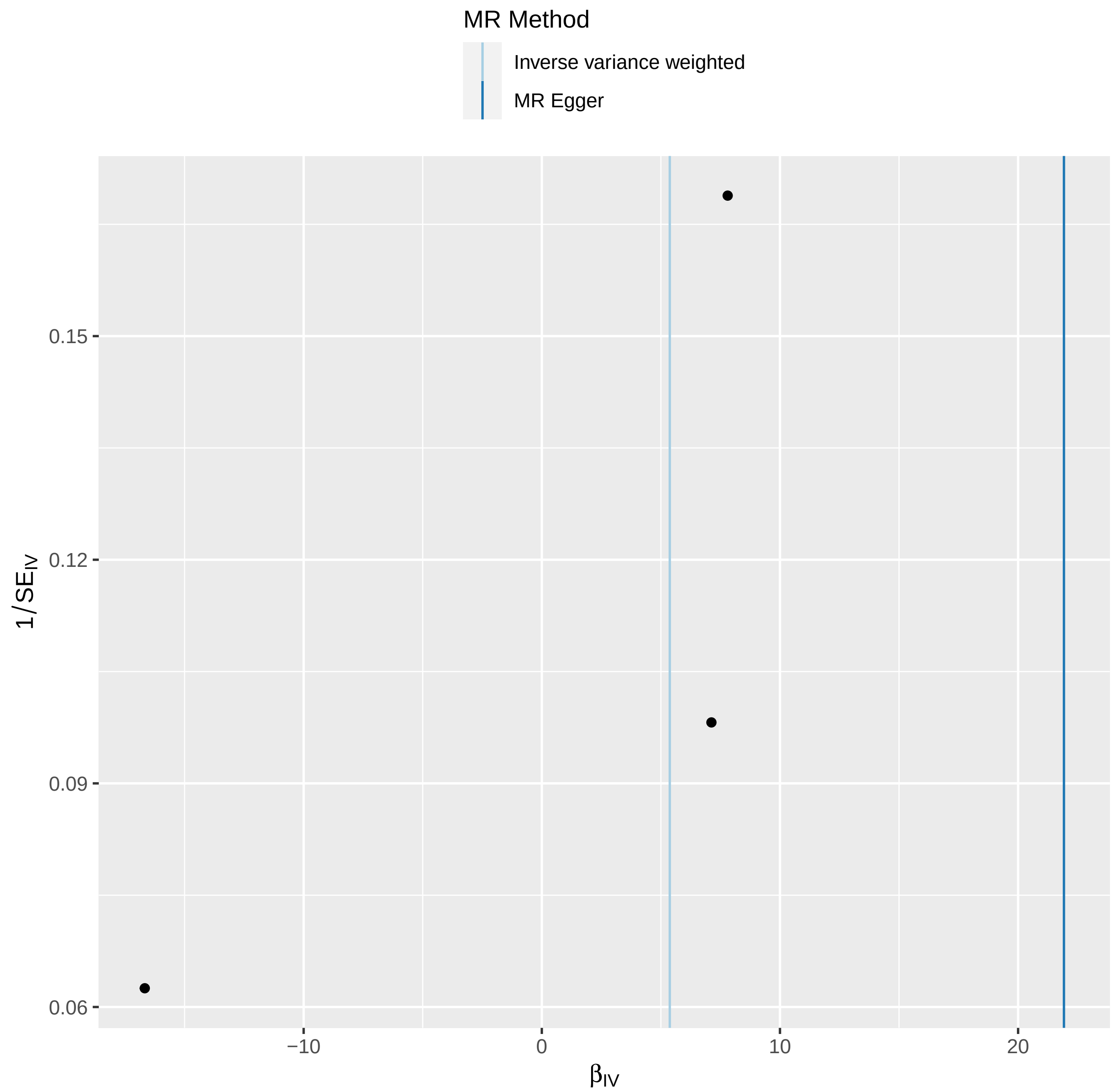

MR Method
Inverse variance weighted
MR Egger
0.15
0.12
0.09
0.06
1/SE$_{IV}$
−10
0
10
20
β$_{IV}$
